# Mating Disruption with Biodegradable Dispensers Complemented with Insecticide Sprays Allows an Effective Management of *Tuta absoluta* in Greenhouse Tomatoes

**DOI:** 10.3390/insects16101035

**Published:** 2025-10-09

**Authors:** Luigi Sannino, Giovanni Benelli, Giulio Piccirillo, Angelo Canale, Andrea Lucchi

**Affiliations:** 1 SESAT Srls, via IX Novembre 15, 81055 Santa Maria Capua Vetere, Italy; luigisannino5@gmail.com (L.S.); giuliopiccirillo@gmail.com (G.P.); 2 Department of Agriculture, Food and Environment, University of Pisa, via del Borghetto 80, 56124 Pisa, Italy; giovanni.benelli@unipi.it (G.B.); andrea.lucchi@unipi.it (A.L.)

**Keywords:** South American tomato pinworm, synthetic sex pheromones, on-farm trials, greenhouse crops, Italy, Integrated Pest Management

## Abstract

**Simple Summary:**

Developing effective tools for the sustainable management of *Tuta absoluta* (Lepidoptera: Gelechiidae) remains a challenge. Pheromone-based control is attracting growing attention for managing this moth pest. In the present study, we assessed the efficacy of a mating disruption approach relying on biodegradable pheromone dispensers, tested at 300 and 500 dispensers/ha, for the control of *T. absoluta* on greenhouse tomato. The mating disruption strategy was coupled with a reference base treatment that alternates the most commonly used insecticides for the pest (the insecticide-based grower’s standard). Mating disruption with biodegradable dispensers achieved a significant reduction in *T. absoluta* male catches and leaf and fruit damages when compared to those obtained with the grower’s standard treatment alone, and performed better than commercial non-biodegradable dispensers. Overall, our study sheds light on the intriguing potential of biodegradable dispensers for *T. absoluta* management on greenhouse tomato.

**Abstract:**

IPM approaches based on pheromone-based techniques for the management of the South American tomato pinworm, *Tuta absoluta* (Meyrick) (Lepidoptera: Gelechiidae), are of great interest. We evaluated the effectiveness of mating disruption (MD) experiments against *T. absoluta* using a biodegradable pheromone dispenser (Isonet-T TT BIOX234) in greenhouse-grown tomatoes over two years in southern Italy. A base treatment alternating the most used insecticides for the pest, i.e., the farmer treatment schedule (FTS), was assigned as a reference, and two MD dispenser densities (i.e., 300 and 500 dispensers/ha) were compared with the MD commercial product Isonet T at 1000 units/ha. We conducted two trials on crops at a density of 37,000 plants/ha. Pest flights were monitored in summer–autumn 2023 and 2024 with pheromone-baited Delta traps. The FTS ensured a generally low level of *T. absoluta* attacks (about 1 leaflet/leaf and 1/300 fruits). Even so, mating disruption resulted in further appreciable reductions in the presence and attacks of the target pest: 89%, 76% and 52% fewer catches; 61%, 45% and 37% fewer mined leaflets; and 76%, 59% and 54% fewer attacked fruits, for Isonet-T TT 500, Isonet-T TT 300 and Isonet T 1000, respectively. Overall, MD biodegradable dispensers could be a valuable tool for controlling *T. absoluta* in greenhouse-grown tomatoes, while also reducing plastic waste in the agricultural setting.

## 1. Introduction

The South American tomato pinworm, *Tuta absoluta* (Meyrick) (Lepidoptera: Gelechiidae), is a major pest of greenhouse and field tomatoes worldwide [1,2,3,4]. Although it can attack a range of species of the Solanaceae family, tomato (*Solanum lycopersicum* L.) is the preferred host [5]. Damage is caused by the larvae, which bore mines on the leaves and galleries inside the fruit and stems. Attacks on leaves cause economic damage only if the number of mines is high, while attacks on fruit almost always result in product waste [1,2,3,4]. In southern Italy, *T. absoluta* is active almost continuously throughout the year, given the mild winter and the widespread presence of protected crops, which facilitate the insect’s activity during the cold season. *T. absoluta* does not enter diapause and temperature has a major impact on its development cycle. Adult flights increase during spring and summer, peak in early autumn, then gradually decrease in October–November and drop sharply in December–January [6]. Without control measures, the pest population remains at a high density throughout most of the second half of the year. In South America, up to a dozen of generations per year have been observed [7]. Along the coastal areas of the Campania region (southern Italy), six to nine overlapping generations occur with life cycles ranging from 40 days for the first and eighth generations, to 28 days in the sixth generation, up to 60 days for the last generation, and 110 days for the overwintering generation [8].

Control of *T. absoluta* is currently achieved through Integrated Pest Management (IPM) approaches, including the use of chemical insecticides, biological control, and agronomic and cultural practices, as well as the use of pheromones for mass trapping and mating disruption [4,9]. Unfortunately, the need to use multiple treatments of synthetic insecticides for managing *T. absoluta* leads to the fast development of resistance in the targeted populations [10,11,12].

In this context, the use of non-chemical insecticide-based management tools, such as biopesticides, parasitoids and predators, along with mass trappings of male moths and pest-resistant plant cultivars can significantly contribute to reducing the risk of resistance development [13,14,15,16,17,18]. Earlier research outlined the efficacy of sex pheromone-based mating disruption (MD) for *T. absoluta* control both in greenhouse and open field tomatoes by using mixtures of the major pheromone component of the moth [19,20,21,22,23,24,25,26] (E,Z,Z)-3,8,11-tetradecatrien-1-yl acetate and the minor component of the pheromone (E,Z)-3,8- tetradecadienyl acetate or just the major component alone [27]. The low rate of parthenogenetic reproduction observed in *T. absoluta* [22] paves the way for the effective use of MD on this moth. However, recent research highlighted the importance of using this strategy as part of an IPM program in order to reduce the possible risk of an increase in the rate of parthenogenesis in target populations [22].

Several studies have been conducted in Mediterranean basin countries testing both hand-applied mating disruption (MD) dispensers (in protected cultivation in southwestern Sardinia, Italy [20], and in heated protected cultivation in the region of Chenchou, El Hamma, Gabes, Tunisia [24,25]) as well as pheromones applied with a caulking gun [23] in an open-field in the Meram district of Konya province, Turkey. In this study, we evaluated the effectiveness of MD with a biodegradable pheromone dispenser over a two-year period on tomatoes grown in greenhouses in southern Italy. As reference, a basic treatment was assigned that alternated the most used insecticides for the pest, i.e., the farmer treatment schedule (FTS), and two different application densities (300/ha and 500/ha dispensers Isonet-T TT BIOX234) were compared with the commercial product MD Isonet T at 1000 dispensers/ha.

## 2. Materials and Methods

### 2.1. Site and Crop Description

Two trials were conducted in the years 2023–2024 in a farm located in the province of Salerno, southern Italy (at Battipaglia, 40°35′04″ N, 15°58′14″ E, 40 m a.s.l., in 2023; at Eboli, 40°34′10″, 15°03′16″, in 2024), where *T. absoluta* attacks are frequently observed from spring to autumn. The host farmer, like most greenhouse tomato producers in the region, controls *T. absoluta* with several insecticide applications, alternating between chemicals and biopesticides (Table 1 and Table 2) at short intervals during the crop cycle. The trial crops of the cultivar Dardo were transplanted on mulched soil on June 28 in 2023 and on May 24 in 2024, at a density of about 37,000 plants/ha. Plants were trained to a single stem fastened to vertical wires. Irrigation and fertilization were performed with a drip system. The crops were terminated on December 10 in 2023 and on October 7 in 2024.

### 2.2. Isonet Devices

The commercial Isonet T and the experimental Isonet-T TT BIOX234, both supplied by Shin-Etsu Chemical Co. Ltd., (Tokyo, Japan), are hand-applied reservoir pheromone dispensers containing the *T. absoluta* sex pheromone [(E,Z,Z)-3,8,11-tetradecatrien-1-yl acetate and (E,Z)-3,8-tetradecadie-yl acetate] [27]. Isonet T is made of plastic polymers and consists of two tubes, one filled with the *T. absoluta* pheromone and the other containing an aluminum wire for the positioning of the dispenser in the greenhouse. Isonet-T TT BIOX234 consists of two parallel capillary tubes of biodegradable polymers joined and sealed at the ends, both filled with the *T. absoluta* synthetic sex pheromone.

### 2.3. Treatments

Four treatments against *T. absoluta* were compared, all including the insecticide spraying program used in the farm (FTS): (1) FTS only; (2) FTS and Isonet T at a density of 1000 units/ha (IT1000); (3) FTS and Isonet-T TT BIOX234 at a density of 300 units/ha (ITX300); (4) FTS and Isonet-T TT BIOX234 at a density of 500 units/ha (ITX500). FTS consisted of 13 sprays in 2023, from July 3 (five days after transplanting) to October 18, and of 9 sprays in 2024, from June 24 (31 days after transplanting) to August 4, alternating between 7 and 10 insecticides (Table 1 and Table 2). The FTS program was aimed at controlling (i) *Frankliniella occidentalis* (Pergande) with the first applications, to reduce the risk of early viral infections, and (ii) various lepidopteran pests, such as *Spodoptera littoralis* (Boisduval), *Helicoverpa armigera* (Hübner) and *Chrysodeixis chalcites* (Esper). Synthetic chemicals (chlorantraniliprole, emamectin benzoate, acibenzolar-s-methyl, cyantraniliprole) were used when an increase in the moth population was expected (July), while *Bacillus thuringiensis*-based preparations were used later, when synthetic chemicals are not permitted.

**Figure 1:**
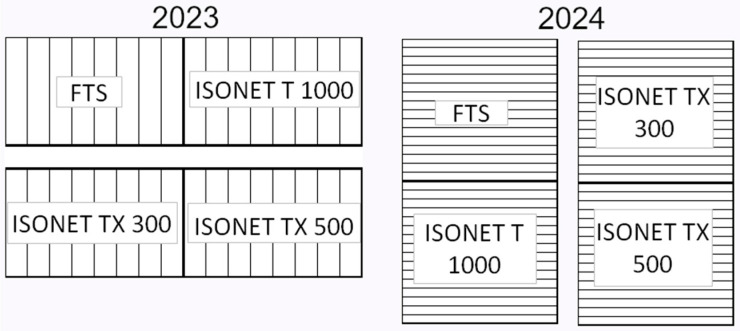
Plot layout of the trials.

Treatments were assigned to unreplicated plots of about 2000 m^2^ in 2023 and 4000 m^2^ in 2024, each consisting of a multi-tunnel cold greenhouse of 8 tunnels in 2023 and 16 tunnels in 2024, with a maximum height of 5 m (Figure 1).

Plots ITX500 and ITX300 were part of a multi-tunnel greenhouse 10 m away from the greenhouse with plots IT1000 and FTS; both greenhouses were fenced on all sides with high transmittance, high greenhouse effect polyethylene film (Lirsalux super type) and insect-proof nets (20 × 10 threads/cm^2^). The plots of the same multi-tunnel (ITX500 and IX300; IT1000 and FTS) were separated only by the fence, which limited air mixing between the two halves of the multi-tunnel. The greenhouses did not have double doors. Ventilation was ensured by openings in the roof, which were also covered by insect-proof nets. Temperature and relative humidity during the trials were monitored with dataloggers (Elitech RC-5, Elitech Technology, Inc., San Jose, CA, USA) placed at 1.5 m height in the greenhouses. The greenhouse complex was bounded by a tomato field of about 25 ha. All Isonet dispensers were placed two days before crop transplanting and evenly distributed according to the application rate by twisting them to the crop support wires at a height of 2 m. The insecticide solutions were prepared shortly before field application and sprayed with a pneumatic atomizer (Caffini 600 L model, Caffini S.p.A., Verona, Italy), using a water volume of 1000 L/ha to ensure a thorough wetting of the plants. After each application, the spray equipment was thoroughly washed and rinsed with water.

### 2.4. Pest Monitoring

The effectiveness of pheromone dispensers in disrupting sexual communication of *T. absoluta* was evaluated by monitoring the flight activity of males and the crop damage.

*Tuta absoluta* male flights were monitored throughout the entire tomato crop cycle, from 26 June to 30 November 2023, and from 22 May to 6 October 2024, with a Biogard Delta trap (BDT) baited with *T. absoluta* pheromone (CBC Biogard, Grassobbio, Italy), placed 1.8 m above the ground in the center of each plot on the same day when Isonet dispensers were placed. Traps were checked weekly, while pheromone dispensers were replaced every 4–5 weeks and sticky trap bottoms changed as needed. Male catches in the disrupted plots were compared with those recorded in the FTS plot.

### 2.5. Leaf and Fruit Damage Estimation

Efficacy assessment was estimated indirectly by counting the number of mined leaflets on two medium-high leaves (20 leaflets/leaf on average) and the number of infested fruits in 25 clusters (six fruits/cluster on average) of three plants for each of ten equally spaced positions along the two central tunnels of the plots. Observations were made at intervals of approximately ten days, eight times in 2023 (every ten days from August 26 to November 4) and six times in 2024 (every ten days from August 17 to October 6), from the appearance of the first symptoms of *T. absoluta* attacks until the end of harvest. Leaflets and fruits were considered damaged if they had at least one epidermis erosion (mine) or hole and were removed after assessment.

### 2.6. Data Analysis

Incidence rates of mined leaflets (number per leaf) and of damaged fruits (number per cluster) were summarized as averages per trial, treatment and sub-plot position. For the inference of average treatment effects (long-run means and related contrasts), a Poisson distribution was fitted to summary counts by treatment and trial, with the logarithm of the parameter as a linear function of treatments and the logarithm of the number of inspected organs as exposure offset, with coefficients starting from a Student distribution covering plausible ranges. Distributions of expected incidence rates per treatment and of related contrasts were computed from simulated distributions of the model coefficients. The analysis was performed with the R programming language [28] and extensions [29,30,31].

## 3. Results

### 3.1. Male Captures

**Figure 2:**
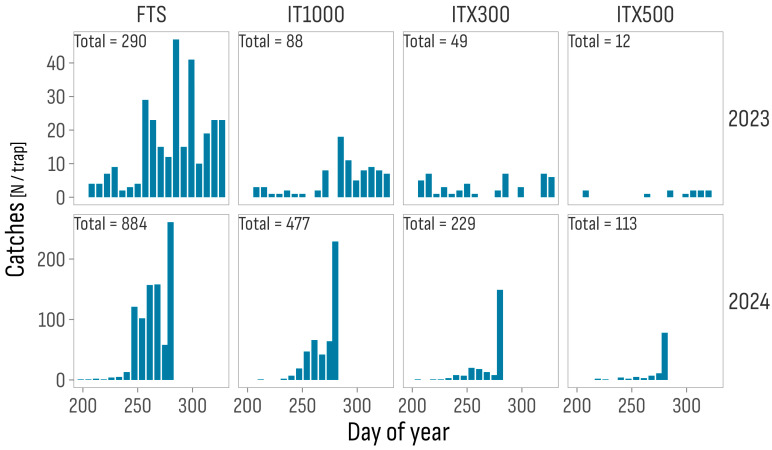
Catches of flying *T. absoluta* adults per day of the year, treatment, and trial year. FTS: Farmer’s treatment schedule of 13 sprays in 2023, from July 3 to October 18, and of 9 sprays in 2024, from June 24 to August 4, with bio- and synthetic insecticides; IT1000: FTS + Isonet T at 1000 pheromone dispensers/ha; ITX300 and ITX500: FTS + Isonet-T TT BIOX234 at 300 and 500 pheromone dispensers/ha.

Catches demonstrated the good specificity of the pheromones used, consisting almost exclusively of *T. absoluta* individuals, with occasional occurrence of the species *H. armigera* and *S. littoralis* (Lepidoptera: Noctuidae). Males were captured from late July to late November in 2023 and from mid-July to early October in 2024, with most catches occurring from mid-September to the second week of November in 2023 and from September to the first week of October, but mostly in the latter week, in 2024. Total catches varied considerably between the different treatments and between the years of testing, with the FTS treatment yielding twice as much as the combined total of the three Isonet treatments in 2023 (290 vs. 149) and slightly more in 2024 (884 vs. 819), a year in which the number of catches was almost four times higher than in 2023 (1703 vs. 439), albeit over a much shorter time interval (Figure 2). Among Isonet treatments, catches were highest for IT1000 and lowest for ITX500 in both years.

### 3.2. Incidence of Mined Leaflets and Damaged Fruits

**Figure 3:**
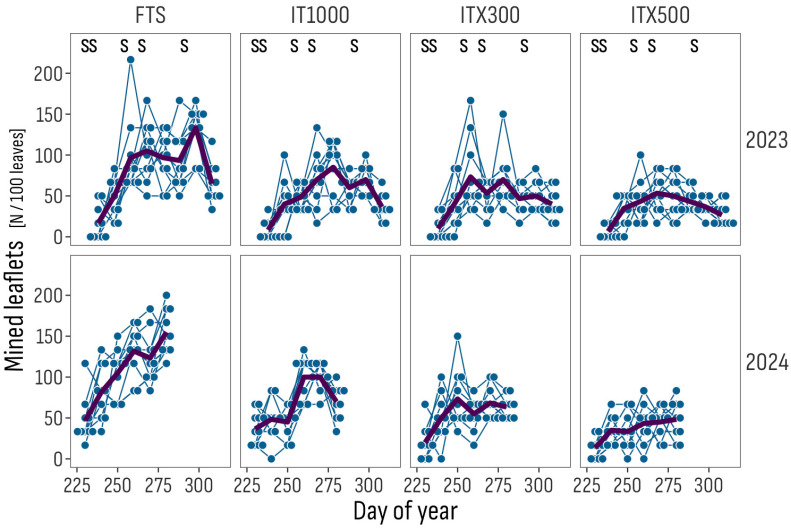
Trend of the incidence of mined leaflets by sub-plot position (gray points and thin lines) and averages (thick lines) per day of the year, treatment and trial year. The S symbol marks occurrences of insecticide sprays. FTS: Farmer’s treatment schedule of 13 sprays in 2023, from July 3 to October 18, and of 9 sprays in 2024, from June 24 to August 4, with bio- and synthetic insecticides; IT1000: FTS + Isonet T at 1000 pheromone dispensers/ha; ITX300 and ITX500: FTS + Isonet-T TT BIOX234 at 300 and 500 pheromone dispensers/ha.

In the 2023 trial, the first signs of the pest attack (mines on leaflets) were observed in late August, on fully developed plants with ripening fruits; the average incidence per 100 leaves was 7 for ITX500, 10 for ITX300, 8 for IT1000 and 17 for FTS (Figure 3). Average incidence of mined leaflets per 100 leaves increased later, moderately for Isonet treatments and considerably for FTS, peaking at 53 for ITX500, 73 for ITX300, 85 for IT1000 and 133 for FTS.

In 2024, the trial started about one month earlier than in 2023 and mined leaflets were first observed toward the end of the second decade of August (day of year: 230), with average incidences per 100 leaves of 13 for ITX500, 20 for ITX300, 37 for IT1000 and 47 for FTS (Figure 3, Supplementary Material Tables S1 and S2). The incidence increased during September, with the number per 100 leaves peaking at 48 for ITX500, 73 for ITX300, 100 for IT1000 and 155 for FTS.

**Figure 4:**
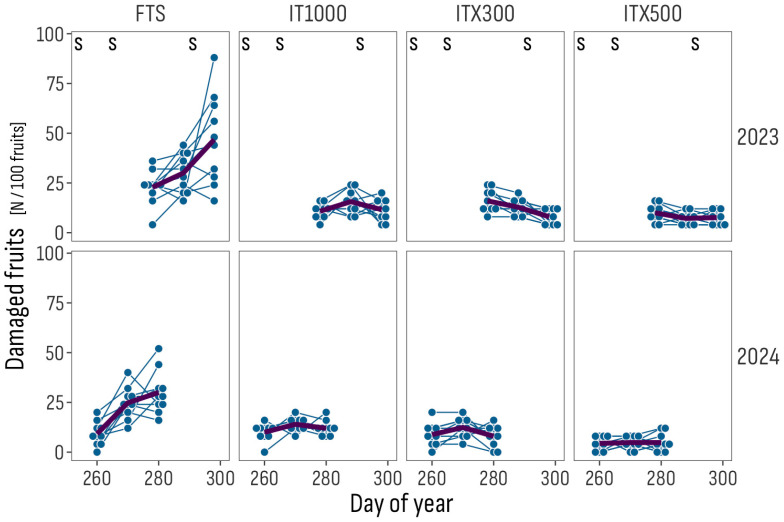
Trend of the incidence of damaged fruits by sub-plot position (points and thin lines) and averages (thick lines) per day of the year, treatment and trial year. The S symbol marks occurrences of insecticide sprays. FTS: Farmer’s treatment schedule of 13 sprays in 2023, from July 3 to October 18, and of 9 sprays in 2024, from June 24 to August 4, with bio- and synthetic insecticides; IT1000: FTS + Isonet T at 1000 pheromone dispensers/ha; ITX300 and ITX500: FTS + Isonet-T TT BIOX234 at 300 and 500 pheromone dispensers/ha.

In both trials, fruit damage symptoms were observed later than leaf mines, by about 40 days in 2023 and 30 days in 2024. No damage by other pests (e.g., *H. armigera*) was detected during the trials. Ranges of average incidences of damaged fruits per 100 clusters in the 2023 and 2024 years were, respectively: 7–10 and 4–5 for ITX500; 8–16 and 8–12 for ITX300; 11–16 and 10–14 for IT1000; 23–47 and 9–30 for FTS (Figure 4).

**Figure 5:**
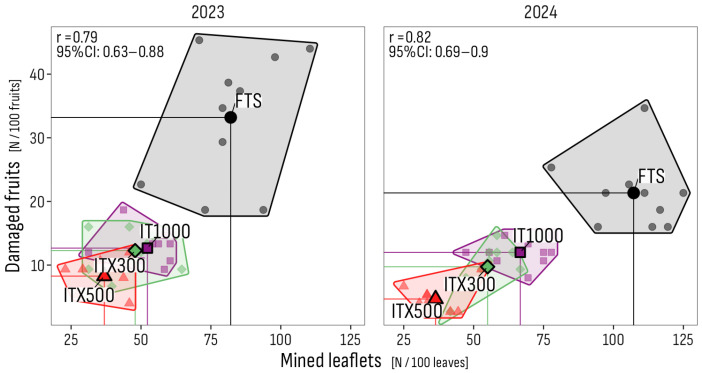
Pearson correlations between average incidences across assessments of mined leaflets and damaged fruits. The correlation across trials was 0.65 (95% CI: 0.5–0.76). Sub-plot positions (points) and averages (circled points marked by treatment names) per treatment and trial year, with enveloping polygons per treatment (FTS: gray; IT1000: pink; ITX300: green; ITX500: red). FTS: Farmer’s treatment schedule of 13 sprays in 2023, from July 3 to October 18, and of 9 sprays in 2024, from June 24 to August 4, with bio- and synthetic insecticides; IT1000: FTS + Isonet T at 1000 pheromone dispensers/ha; ITX300 and ITX500: FTS + Isonet-T TT BIOX234 at 300 and 500 pheromone dispensers/ha.

Overall, the average incidences of leaf and fruit damage were low, not exceeding three leaflets on two leaves and one fruit on two clusters, as might be expected from the uniform schedule of frequent sprays, which ended two weeks before the first observation in 2024, while covering most of the observation interval in 2023. Nevertheless, in both years, a significant reduction in infestation was achieved with MD devices, with Isonet-T TT BIOX234 achieving a greater reduction than Isonet T (Figure 5).

### 3.3. Relationship Between Male Catches and Damages

**Figure 6:**
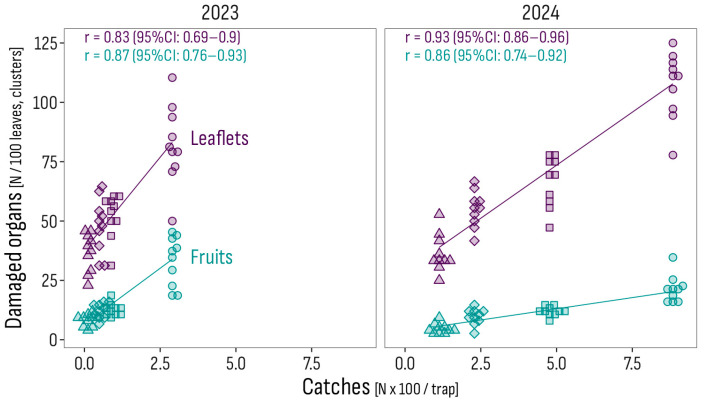
Pearson correlations between the number of catches and the incidence of mined leaflets and of damaged fruits per trial year. The correlations across trials were 0.82 (95% CI: 0.74–0.88) for the incidence of mined leaflets and 0.39 (95% CI: 0.19–0.56) for the incidence of damaged fruits. Treatments marked by symbols: FTS (circle); IT1000 (square); ITX300 (diamond); ITX500 (triangle). FTS: Farmer’s treatment schedule of 13 sprays in 2023, from July 3 to October 18, and of 9 sprays in 2024, from June 24 to August 4, with bio- and synthetic insecticides; IT1000: FTS + Isonet T at 1000 pheromone dispensers/ha; ITX300 and ITX500: FTS + Isonet-T TT BIOX234 at 300 and 500 pheromone dispensers/ha.

In both years, the incidence of leaf and fruit damage was positively correlated with the total frequency of male catches, with 100 more catches corresponding to 16 more mined leaflets per 100 leaves and 9 more damaged fruits per 100 clusters in 2023, and 9 more mined leaflets and 2 more damaged fruits per 100 leaves and clusters, respectively, in 2024 (Figure 6).

### 3.4. Expected Values

**Figure 7:**
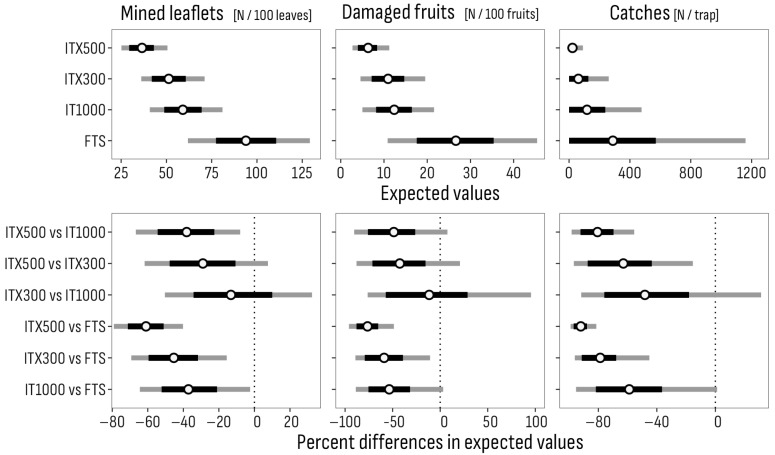
Central positions (circular points) and 80% (black bars) and 95% (gray bars) highest-density intervals of expected values and of related pairwise percent differences of the incidence of mined leaflets and damaged fruits and of the number of captures of flying adults per treatment (long-run means for identical experimental conditions). FTS: Farmer’s treatment schedule of 13 sprays in 2023, from July 3 to October 18, and of 9 sprays in 2024, from June 24 to August 4, with bio- and synthetic insecticides; IT1000: FTS + Isonet T at 1000 pheromone dispensers/ha; ITX300 and ITX500: FTS + Isonet-T TT BIOX234 at 300 and 500 pheromone dispensers/ha.

The expected values (long-run means for the trial conditions) of the incidence of mined leaflets and of damaged fruits per treatment, as well as the percent differences in expected values between pairs of treatments, are represented in Figure 7 by 80% and 95% credible intervals of plausible values.

The credible intervals for the ITX300 and the IT1000 treatments overlap with their upper half at the lower part of those for the FTS treatment, while the interval for ITX500 is lower and completely outside. The expected values for male catches differ similarly between treatments, though the distributions of plausible values all overlap toward low values (Figure 7).

Plausible values for the percentage differences in expected incidence values between the Isonet treatments and the reference FTS are well in the negative side for both mined leaflets and damaged fruits, and male catches, with intervals of incidence reductions between zero and 80% for mined leaflets and between zero and 90% for damaged fruits and male catches. Among the Isonet treatments, the differences in the plausible percentage intervals between ITX300 and IT1000 are mostly in the negative range for the incidence of mined leaflets, damaged fruits and male catches, but also with portions of positive values, particularly for fruit damage, showing a slightly higher expected efficacy for ITX300. Intervals for differences between ITX500 and ITX300 show a moderately higher expected efficacy in leaf and fruit damage control and a higher efficacy in male captures for ITX500, while the efficacy of ITX500 is expected to be generally higher than IT1000.

## 4. Discussion

Although the use of MD products against *T. absoluta* has become increasingly popular in recent years in several regions of the world [4,32,33], to our knowledge, this is the first research focusing on biodegradable dispensers. The trial site offers very favorable conditions for *T. absoluta,* with a mild climate and large areas cultivated in cold plastic greenhouses, which allow harmful populations of the pest to develop and remain active during the cold season [8], while the natural enemies of this moth are limited by widespread intensive growing conditions (e.g., large amounts of chemicals). Given the need for large plots for disruption trials, a treatment with Isonet devices alone (without insecticide interventions) or with on-site replications on the commercial farm hosting the trial was not possible. However, similar results obtained in the two-year trial with Isonet, under different seasonal conditions and pest population densities, showed a remarkable enhancement in control efficacy compared to the spraying program alone.

In both years, male captures increased in the second half of the crop cycle (from mid-September in 2023 and from late August in 2024), probably due to the non-use of chemical sprays during harvest. The lower density of *T. absoluta* population in the 2023 trial could have been due in part to seasonal fluctuations, but mostly to strict control practices in the surrounding area of intensive, protected vegetable cultivation, leading to a higher containment of the pest population in the environment, compared to the more diversified agricultural landscape of the 2024 trial location. The frequency of catches in Isonet plots was consistently lower than in the FTS plot, with the lowest values and no catches in the first two months of monitoring for ITX500 compared to ITX300 and IT1000. Damage levels were positively correlated with total catches. The incidence of damage was significantly higher in 2023 than in 2024, probably due to later transplanting (June 28 vs. May 24) that exposed the crop to the pest when its population peaks in the area [6]. The FTS treatment ensured a low level of *T. absoluta* damage, averaging less than one leaflet per leaf and less than one fruit in 350 clusters, with larval activity ending before the end of adult flights. This level of leaf and fruit damage was further reduced with Isonet treatments, respectively, by 37% and 55% with IT1000, by 45% and 60% with ITX300, and by 61% and 76% with ITX500. Isonet T has been already tested in southern Italy at rates of 600 and 1000 dispensers/ha, also with promising control effectiveness of *T. absoluta* [34], but when applied at the higher dose in our trials it was always outperformed by Isonet-T TT BIOX234, consistently at 500 and, to a lesser extent, at 300 dispensers/ha. The positive performance of Isonet treatments could be partly due to the low density of the pest population, thanks to the knockdown effect of early insecticide sprays (spinosad, chlorantraniliprole), which prevented the population from developing.

The control levels obtained with Isonet T at the rate of 1000 dispensers/ha agree with those of similar trials conducted in southwestern Sardinia (Italy), where reductions of 93–97% in male catches, 57–85% in leaf damage, and 62–89% in fruit damage were observed in disrupted greenhouses, compared to the control [20]. Tested at rates of 500, 750 and 1000 dispensers/ha in protected tomato crops in Kuwait, Isonet T was as effective at the highest rate as the insecticide control program based on three applications of flubendiamide, in May–July, and six applications, first with flubendiamide and then with azadirachtin, in December–February (flubendiamide: 60 mg L^−1^; azadirachtin: 3 g L^−1^) [21].

In a heated greenhouse in Tunisia, Isonet T applied alone at a rate of 1000 dispensers/ha showed control rates comparable to those obtained when combined with monthly bioinsecticide sprays (based on *B. thuringiensis* ssp. *kurstaki* and *Azadirachta indica* A.Juss., at rates of 3 kg-L/ha) and to those obtained with a specialized pheromone and lure application technology (SPLAT TUTA). The latter involves the use of a pheromone gel mixed with the insecticide spinosad at the rate of 17 mL/kg of pheromone (attract-and-kill technique), applied weekly at the rate of 1 g/point to the axil of an upper leaf once the plant had reached 1 m in height [25]. On the other hand, TUTATEC passive dispensers of the sex pheromone mixture (E,Z,Z)-3,8,11-tetradecatrienyl acetate and (E,Z)-3,8-tetradecadienyl acetate (SEDQ Healthy Crops SL, Barcelona, Spain), applied at 600 dispensers/ha (111 g a.i. of sex pheromone/ha) to an early spring tomato crop in heated greenhouses in southern Tunisia, reduced leaf and fruit infestation by 29% and 36%, respectively, compared to 32% and 72% obtained with insecticide control alone [24]. The latter consisting of two applications with azadirachtin (10 g/L) and one application with cyantraniliprole (100 g/L) at doses of 3 L/ha and 0.6 L/ha, respectively.

In Spain, Vacas et al. (2011) [19] evaluated mesoporous pheromone dispensers at different doses (10 to 40 g a.i./ha), emitted at a constant rate over four months, finding that MD provided pest control levels comparable to that obtained with chemical control alone (three applications of indoxacarb 30% at doses of 100 g/ha, alternated with one application of spinosad 480 g/L at the dose of 300 g/ha), only when MD was applied in a greenhouse protected by a double door and a fine mesh net. In a two-year experiment conducted in Turkey (Konya province) under open-field conditions, where the weather influence on pheromone dispersal is higher and the attraction of adults from the surrounding areas is continuous, application of the SPLAT TUTA technology with a pheromone gel at 2500 g/ha reduced *T. absoluta* infestation by 63% and 75% [23]. Recently, in an experiment conducted on protected tomato crops in Ningcheng, Inner Mongolia and in Youjun Town, Sichuan, both passive polyethylene tube dispensers and active pheromone aerosol devices at various rates showed a significant reduction in damage rates and larval populations [26]. 

In conclusion, mating disruption applied with biodegradable pheromone dispensers can play a useful role in protecting greenhouse tomato crops from *T. absoluta*, allowing a reduction in insecticide sprays and related residues in products, with costs partially offset by the reduction in insecticide application costs. Protected cultivations are particularly suitable for the mating disruption technique and there is ample prospect for its application in table tomatoes, which are mostly produced in greenhouses.

## Figures and Tables

**Table 1:** Formulates employed for managing *Tuta absoluta* in the field trials.

Formulate	Active Substance	Formulation	Concentration (%)
ISONET-T TT BIOX234	(E,Z,Z)-3,8,11-tetradecatrien-1-yl acetate;(E,Z)-3,8-tetradecadien-1-yl acetate	VP *	55–75% 5–8%
ISONET T	(E,Z,Z)-3,8,11-tetradecatrien-1-yl acetate;(E,Z)-3,8-tetradecadien-1-yl acetate	VP *	58.2–68.2% *w*/*w* 5.4–8.4% *w*/*w*
DICARZOL 50 SP **	Formetanate	SP	10.5
INTREPID	Methoxyfenozide	SC	240 g/L
MINECTO ALPHA	Acibenzolar-s-methyl, cyantraniliprole	SC	12.5 g/L, 100 g/L
SIMPELL/LASER	Spinosad	CS	44.2%
AFFIRM	Emamectin benzoate	SG	0.95%
ALTACOR	Chlorantraniliprole	WG	35%
VERTIMEC EC	Abamectin	EC	1.84%
TUREX	*Bacillus thuringiensis* ssp. *kurstaki* & *aizawai*	WP	50%
PRIMIAL	*Bacillus thuringiensis* ssp. *kurstaki* st. SA11	WG	6.4%
COSTAR WG	*Bacillus thuringiensis* ssp. *kurstaki* st. SA12	WG	18% *w*/*w*
AGREE WG	*Bacillus thuringiensis* ssp. *aizawai* st. GC91	WG	50%

* Vapor-releasing product. ** Not authorized in Italy against *T. absoluta*, but with known partial activity against the pest. In this case used only at the start of the trial for control of the western flower thrips *Frankliniella occidentalis* (Pergande).

**Table 2:** Details of the farmer’s treatment schedules carried out in this study.

Trial 2023			Trial 2024		
Day	Formulate	Rate, g-mL/hL	Day	Formulate	Rate, g-mL/hL
July-03	DICARZOL 50 SP	125	June-24	PRIMIAL, TUREX	150, 200
July-10	DICARZOL 50 SP	125	July-01	SIMPELL, LASER	20, 20
July-20	LASER	20	July-09	ALTACOR	12
July-24	COSTAR WG, AGREE WG	200, 200	July-10	PRIMIAL, TUREX	150, 200
July-25	AFFIRM	150	July-18	AFFIRM	150
July-31	AGREE WG	200	July-19	PRIMIAL, TUREX	150, 200
August-04	LASER, INTREPID	25, 50	July-21	VERTIMEC	60
August-10	AGREE WG	200	July-23	PRIMIAL, TUREX	120, 200
August-18	MINECTO	100	August-04	PRIMIAL, AGREE	150, 200
August-23	AGREE WG	200			
September-11	AGREEWG	200			
September-22	COSTAR WG	200			
October-18	COSTAR WG	200			

## Data Availability

Data are available in the manuscript; the whole dataset can be requested to the authors.

## Supplementary Materials

The following supporting information can be downloaded at: https://www.mdpi.com/article/10.3390/insects16101035/s1, Table S1: Percent reduction of the incidence of mined leaflets and damaged fruits with mating disruption (MD) vs. the insecticide spraying program used in the farm (FTS) by treatment and observation in the 2023 trial; Table S2: Percent reduction of the incidence of mined leaflets and damaged fruits with mating disruption (MD) vs. the insecticide spraying program used in the farm (FTS) by treatment and observation in the 2024 trial.
