# Supplementary material for: Mating Disruption with Biodegradable Dispensers Complemented with Insecticide Sprays Allows an Effective Management of Tuta absoluta in Greenhouse Tomatoes
Source: Insects. 2025 Oct 9;16(10):1035. doi: 10.3390/insects16101035 (PMC12564165; doi:10.3390/insects16101035)
Supplement: Supplementary file 1 [file insects-16-01035-s001.zip › insects-3895618-supplementary.pdf]

Supplementary Material

Table S1. Percent reduction of the incidence of mined leaflets and damaged fruits with mating disruption (MD) vs the insecticide spraying program used in the farm (FTS) by treatment and observation in the 2023 trial.

Treatment	Aug-26	Sep-05	Sep-15	Sep-25	Oct-05	Oct-15	Oct-25	Nov-04	Average
					Mined leaflets				
IT1000	50	20	50	33	12	36	47	44	37
ITX300	40	20	24	49	28	50	62	38	39
ITX500	60	30	55	49	48	54	72	59	53
					Damaged fruits				
IT1000					53	48	75		59
ITX300					30	57	83		57
ITX500					56	76	84		72

Table S2. Percent reduction of the incidence of mined leaflets and damaged fruits with mating disruption (MD) vs the insecticide spraying program used in the farm (FTS) by treatment and observation in the 2024 trial.

Treatment	Aug-17	Aug-27	Sep-6	Sep-16	Sep-26	Oct-6	Average
				Mined leaflets			
IT1000	21	41	57	24	19	55	36
ITX300	57	39	30	58	45	59	48
ITX500	71	57	68	67	64	69	66
				Damaged fruits			
IT1000				-9	44	60	32
ITX300				4	50	73	43
ITX500				52	81	84	72
